# Supplementary material for: Asymmetric Synthesis of Quaternary Hydantoins via a Palladium-Catalyzed Aza-Heck Cyclization
Source: J Am Chem Soc. 2025 Nov 14;147(49):44692–8. doi: 10.1021/jacs.5c16022 (PMC12703750; doi:10.1021/jacs.5c16022)
Supplement: Supplementary file 2 [file ja5c16022_si_002.zip › All NMR FID Files/24/24_AllNMR/1/pdata/1/email_MLM03110-5_1_1.pdf]

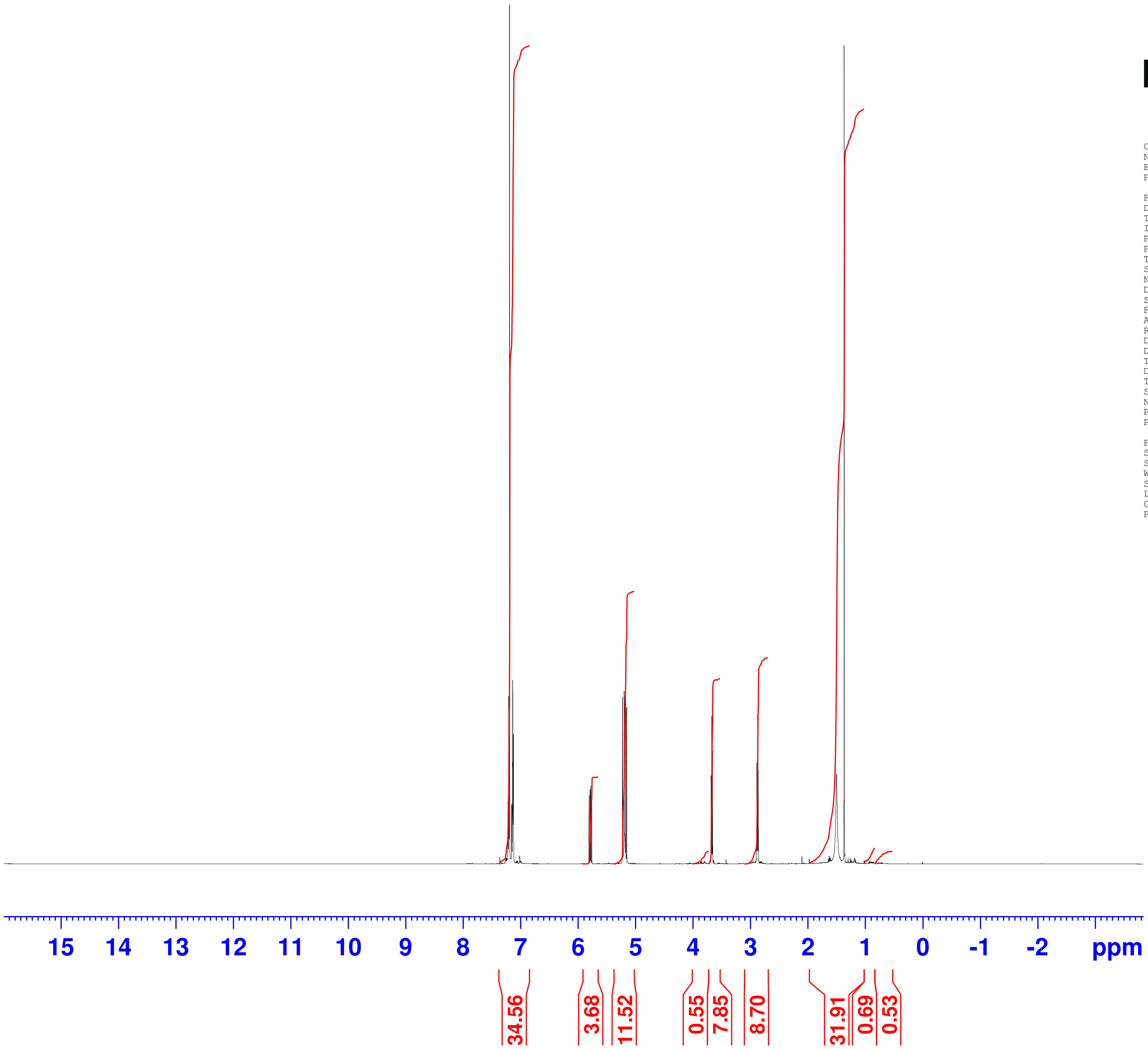

BRUKER

```
Current Data Parameters
NAME          MLM03110-5
EXPNO                  1
PROCNO                 1

F2 - Acquisition Parameters
Date_           20250714
Time               21.06 h
INSTRUM  Avance Neo 600 at University of Delaware
PROBHD   Z185170_0001 (
PULPROG               zg
TD                 65536
SOLVENT            CDCl3
NS                    16
DS                     2
SWH            11904.762 Hz
FIDRES          0.363304 Hz
AQ             2.7525120 sec
RG                    16
DW                42.000 usec
DE                 10.61 usec
TE                 298.0 K
D1           10.00000000 sec
TD0                    1
SFO1         600.1337058 MHz
NUC1                  1H
P1                  8.07 usec
PLW1          6.90000010 W

F2 - Processing parameters
SI                 65536
SF           600.1300569 MHz
WDW                   EM
SSB                    0
LB                  0.30 Hz
GB                     0
PC                  1.00
```
